# Endovascular Stent‐Graft Exclusion for the Treatment of Haemoptysis Caused by Central Pulmonary Artery Pseudoaneurysm: A Case Report

**DOI:** 10.1002/rcr2.70714

**Published:** 2026-08-04

**Authors:** Dongjun Su, Shuangxi Li, Fenqiang Li

**Affiliations:** ^1^ Interventional Department Lanzhou University First Affiliated Hospital Lanzhou Gansu China

**Keywords:** endovascular exclusion, haemoptysis, pulmonary artery pseudoaneurysm, stent‐graft

## Abstract

Pulmonary artery pseudoaneurysm (PAP) is a rare lethal disease; central tuberculous PAP carries a mortality rate of 50%–90% and often responds poorly to conventional embolization. We report a 59‐year‐old tuberculous woman with recurrent haemoptysis secondary to left main PAP. Covered stent‐graft exclusion was conducted after failed conservative treatment and bronchial artery embolization, with complete haemostasis and no recurrence at 1‐year follow‐up. Most existing literature adopts vessel‐occlusive embolization for PAP, which may cause pulmonary infarction. Moreover, stent‐graft implantation for main pulmonary artery lesions is seldom reported due to dimensional mismatch between large native main pulmonary arteries and commercially available covered stent‐grafts (maximum diameter 12 mm). Our case uniquely confirms that precise preoperative vascular measurement allows successful deployment of a 12 mm stent‐graft within the 11 mm proximal arterial segment, providing rare long‐term follow‐up evidence of durable haemostasis and preserved pulmonary patency via this vessel‐preserving intervention strategy for central PAP.

## Graphical abstract

Emergency stent‐graft exclusion effectively cured life‐threatening haemoptysis from central pulmonary artery pseudoaneurysm refractory to conventional therapy, with sustained haemostasis during 1‐year follow‐up, supporting this minimally invasive strategy as a definitive treatment option.

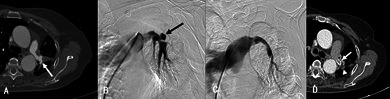

## 1 Introduction

Pulmonary artery pseudoaneurysm (PAP) is a rare fatal vascular complication with a mortality rate of 50%–90% after rupture [1]. Central PAP involving the main pulmonary artery is difficult to treat and associated with poor prognosis. Bronchial artery embolization and aneurysmal sac embolization irreversibly impair pulmonary arterial perfusion, which may lead to distal hypoperfusion, dyspnea, pulmonary infarction and long‐term pulmonary dysfunction; hence these approaches are unsuitable for lesions located in the main pulmonary artery [2].

Endovascular exclusion with covered stent‐grafts represents a novel vessel‐preserving strategy for high‐risk central PAP. It isolates the pseudoaneurysm to halt active bleeding while preserving the native main pulmonary artery and distal pulmonary circulation, thereby avoiding ischemic complications induced by conventional embolization. Nevertheless, reports on stent‐graft implantation for main pulmonary artery PAP remain scarce due to the low disease incidence and limited sizes of commercially available covered stent‐grafts.

We herein report a patient with tuberculous left main PAP presenting with recurrent massive haemoptysis. Haemoptysis persisted after prior bronchial artery embolization at an external hospital, and complete haemostasis was achieved after covered stent‐graft implantation without recurrence during the 1‐year follow‐up. This article summarizes the clinical features, key interventional techniques and long‐term outcomes of this vessel‐preserving stent‐graft therapy and provides clinical evidence for individualized minimally invasive management of central PAP.

## 2 Case Report

**FIGURE 1:**
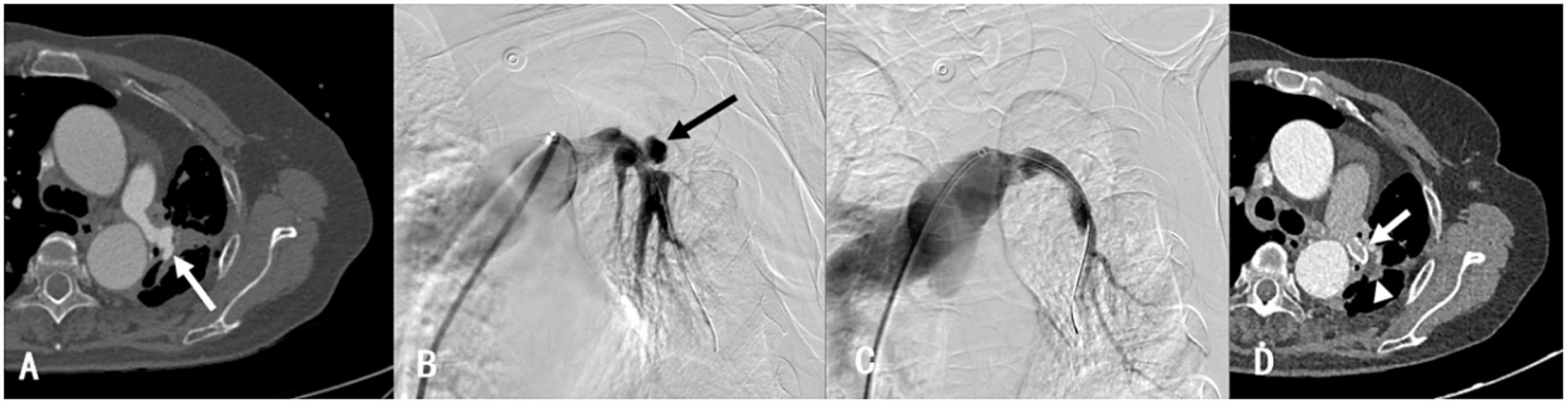
(A) Enhanced CTPA shows a saccular contrast agent filling shadow in the left main pulmonary artery. (B) Left main pulmonary artery angiography shows opacification of the pseudoaneurysm (black arrow). (C) Angiography after endovascular exclusion with covered stent shows no opacification of the pseudoaneurysm sac. (D) Chest enhanced CT at 1 year after surgery shows that the stent in the left main pulmonary artery is in good position with smooth blood flow and no opacification of the original pseudoaneurysm.

A 59‐year‐old female patient was admitted to our hospital due to intermittent bright‐red haemoptysis for 20 days without obvious predisposing factors, with a maximum of 3–5 mL bright‐red blood per episode. Despite conservative medical management and bronchial artery embolization performed at a referring hospital (targeting bilateral bronchial arteries and the right intercostobronchial artery), haemoptysis remained incompletely controlled, with an estimated total blood loss of approximately 100 mL. The referring hospital first performed bronchial artery embolization, as the initial emergency computed tomography (CT) failed to detect the PAP, and clinicians initially presumed that the recurrent haemoptysis stemmed from bronchial arterial lesions. On admission, physical examination revealed a body temperature of 36.5°C, blood pressure of 139/97 mmHg, heart rate of 97 beats/min, respiratory rate of 20 breaths/min and blood oxygen saturation of 90% without oxygen supplementation. Laboratory examinations showed a white blood cell count of 14.68 × 10^9^/L, haemoglobin of 131 g/L, platelet count of 160 × 10^9^/L; coagulation profile: prothrombin time 13.3 s, activated partial thromboplastin time 25.1 s, fibrinogen 1.87 g/L, D‐dimer 0.62 mg/L (normal < 0.5 mg/L). Sputum acid‐fast staining, bacterial culture, and *Mycobacterium tuberculosis* and drug‐resistance gene tests were negative. Contrast‐enhanced chest CT and computed tomographic pulmonary angiography (CTPA) demonstrated an irregular pseudoaneurysm of the left main pulmonary artery (Figure 1A).

The patient had a 16‐year history of pulmonary tuberculosis with standard anti‐tuberculosis treatment, leaving multiple nodules, calcifications and fibrous streaks in both lungs; she also had a 5‐year history of hypertension with regular medication. After admission, intravenous haemostatic therapy with pituitrin and phentolamine was administered, but intermittent haemoptysis persisted. Given the imaging confirmation of PAP and stenosis of the left main pulmonary artery, as well as the potential recanalization of embolized bronchial and non‐bronchial systemic arteries at the outside hospital, we planned to perform embolization of the culprit arteries first, followed by stent‐graft exclusion for definitive haemostasis.

The right femoral artery was punctured using the Seldinger technique. Based on the feeding vessel evaluation via contrast‐enhanced chest CT, superselective catheterization and embolization were sequentially performed on the left bronchial artery, right intercostobronchial artery and bilateral inferior phrenic arteries with 300–500 μm polyvinyl alcohol (PVA) particles. Catheter angiography of bilateral subclavian arteries revealed no ectopic bronchial arteries or abnormal non‐bronchial systemic arteries. During the procedure, the patient developed sudden massive haemoptysis (approximately 400 mL in a single episode), which was considered directly related to the PAP. We immediately switched to right femoral vein puncture and inserted an 8F introducer sheath. Under the assistance of a 5F single‐curved catheter, a 260 cm stiff guidewire was advanced across the pseudoaneurysm of the left main pulmonary artery into the distal pulmonary artery branches. Angiography through an 8F long sheath confirmed the pseudoaneurysm of the left main pulmonary artery (Figure 1B).

Preoperative CT measurements showed that the diameters of the normal vessels proximal and distal to the aneurysm were 11 and 10.5 mm, respectively. A Bard Fluency Plus 12 × 40 mm balloon‐expandable stent‐graft (ePTFE‐covered) was selected and precisely deployed under road‐mapping guidance. Follow‐up angiography showed clear opacification of the distal pulmonary artery branches and no opacification of the left main PAP sac (Figure 1C).

The patient expectorated old blood clots within 2 days after surgery. Haemoptysis completely resolved before discharge, with a haemoglobin level of 113 g/L at discharge. At the 1‐year follow‐up, there was no recurrence of haemoptysis. Contrast‐enhanced chest CT showed a well‐positioned stent with patent blood flow, and complete thrombosis of the original pseudoaneurysm (Figure 1D).

## 3 Discussion

In patients with chronic pulmonary tuberculosis, PAP represents the leading source of pulmonary artery‐originated haemoptysis. This disease is easily missed clinically, and once ruptured after misdiagnosis, the mortality rate reaches 50%–100%. Its etiologies are diverse, including infection, iatrogenic injury, trauma, vasculitis and malignant tumours [3].

CTPA is the standard imaging modality for diagnosing PAP, which can identify the presence, location, size, morphology of the lesion, and its anatomical relationship with adjacent vessels, as well as evaluate underlying lung diseases and related complications. In this case, CTPA showed typical imaging features of old pulmonary tuberculosis around the pseudoaneurysm, providing key evidence for etiological diagnosis [4]. The core of treatment for PAP is to prevent rupture and bleeding. Current mainstream treatments include CT‐guided percutaneous aneurysm sac embolization with tissue glue, surgical resection and endovascular therapy.

Endovascular therapy has gradually become the first‐line treatment due to its minimal invasiveness and low complication risk. It mainly consists of vessel‐occlusive embolization and vessel‐preserving covered stent‐graft exclusion, alongside other less common interventional modalities. Coil or glue embolization achieves haemostasis by occluding the feeding artery of the aneurysm, yet this approach inevitably blocks normal pulmonary artery branches, which may further result in pulmonary infarction, persistent hypoxemia and deteriorated pulmonary function. In contrast, covered stent‐grafts are capable of sealing the aneurysm sac while preserving the patency of the main pulmonary trunk and distal pulmonary circulation. This technique is particularly indicated for pseudoaneurysms arising from the main pulmonary artery trunk, as permanent vessel occlusion of such lesions would severely compromise pulmonary perfusion. Preoperative measurement of pulmonary artery diameter serves as the fundamental criterion for stent selection. In this single case, the 12‐mm stent‐graft adequately matched the 11‐mm vessel, achieving complete exclusion of the pseudoaneurysm without obvious stent oversizing at both ends, which provides preliminary experience for similar cases [2, 5]. The failure of previous bronchial artery embolization further illustrates its limitation: it merely addresses systemic collateral supply rather than the primary pulmonary arterial rupture, thereby mandating covered stent‐graft exclusion for definitive haemostasis.

Reports of covered stent‐graft repair for main PAP remain limited, especially among patients with tuberculous lesions, mainly due to the extremely low incidence of the disease and the lack of dedicated pulmonary artery stent‐grafts: the diameter of the left and right main pulmonary arteries in adults is mostly 14–26 mm, while the maximum diameter of clinically available stent‐grafts is only 12 mm. The successful implementation of stent‐graft treatment in this patient benefited from the precise preoperative measurement of the proximal aneurysm diameter (11 mm), which matched the 12 mm stent‐graft, and our centre's 11 years of clinical experience in endovascular treatment of PAP. Haemoptysis was immediately controlled after surgery, and there was no recurrence at the 1‐year follow‐up, confirming the definitive efficacy.

The choice between transcatheter embolization and covered stent‐graft repair is determined by the perfusion significance of the target vessel. Selective embolization constitutes the first‐line therapy for non‐essential visceral branch arteries, such as the internal thoracic, splenic arteries [6, 7]. In contrast, covered stent‐grafts are indicated for major vascular trunks to preserve distal organ perfusion, exemplified by the main pulmonary artery and intracranial arterial trunks. This principle aligns with the treatment strategy adopted for the PAP in our case [8].

Endovascular stent‐graft exclusion is a safe and effective minimally invasive treatment option for patients with pulmonary tuberculosis complicated by ruptured central PAP. High‐risk patients can achieve an excellent prognosis through precise stent positioning, appropriate stent selection and individualized perioperative management. Clinicians should be highly alert to sudden massive haemoptysis in patients with pulmonary tuberculosis and promptly perform CTPA to screen for this treatable critical complication. Long‐term standard anti‐tuberculosis treatment and regular imaging follow‐up are crucial for preventing recurrence.

## Author Contributions

**Dongjun Su:** conceptualization, data curation, formal analysis, investigation, methodology, writing – original draft, writing – review and editing. **Shuangxi Li:** data curation, investigation, methodology, validation, visualization. **Fenqiang Li:** supervision, conceptualization, project administration, resources, writing – review and editing, funding acquisition.

## Funding

This study was supported by the Natural Science Foundation of Gansu Province (no. 24JRRA303).

## Consent

The authors declare that written informed consent was obtained for the publication of this manuscript and accompanying images using the consent form provided by the journal.

## Conflicts of Interest

The authors declare no conflicts of interest.

## Data Availability

The data that support the findings of this study are available on request from the corresponding author. The data are not publicly available due to privacy or ethical restrictions.
